# Feature-based clustering of hemoglobin trajectories and subsequent risk of end-stage kidney disease in stages 3–4 CKD: a landmark analysis

**DOI:** 10.3389/fnut.2026.1830364

**Published:** 2026-05-07

**Authors:** Na Sun, Nan Hu, Jinping Li, Xichao Wang, Wenyu Zhang, Shunya Uchida, Yingying Han, Wenxiu Chang

**Affiliations:** 1 Department of Nephrology, Tianjin First Central Hospital, Tianjin, China; 2 Department of Medicine, Teikyo University School of Medicine, Tokyo, Japan

**Keywords:** chronic kidney disease, dialysis, hemoglobin, nutritional status, prognosis, protein-energy wasting, trajectory analysis

## Abstract

**Background:**

Anemia and protein-energy wasting (PEW) are common complications in chronic kidney disease (CKD) and are associated with disease progression. However, the prognostic significance of longitudinal hemoglobin (Hb) trajectory patterns, particularly in the context of nutritional status, remains unclear. This study aimed to identify distinct Hb trajectories in CKD stages 3–4 patients and evaluate their associations with renal outcomes, employing a dual analytical approach: exploratory analysis of concurrent eGFR decline and predictive analysis of subsequent dialysis risk using a landmark design.

**Methods:**

This retrospective landmark cohort study included 694 non-dialysis CKD patients from a Japanese medical center. All completed a 2-year landmark period with Hb measured at regular 90-day intervals (± 15 days). Feature-Based Clustering of Longitudinal Trajectories was applied to Hb measurements during the 2-year landmark period to identify trajectory groups. For exploratory association analysis, Cox proportional hazards regression assessed the relationship between Hb trajectories and concurrent ≥30% eGFR decline within the landmark period. For predictive risk stratification, competing risk Fine-Gray regression models (with death as the competing event) evaluated the risk of dialysis initiation occurring after the 2-year landmark period, with trajectory groups serving as baseline predictors.

**Results:**

Five distinct Hb trajectory groups were identified: (1) Low-Stable (*n* = 181, 26.1%); (2) High-Stable (*n* = 157, 22.6%); (3) Rapid-Declining (*n* = 124, 17.9%); (4) Lowest-Plateau (*n* = 122, 17.6%); and (5) Low-Declining (*n* = 110, 15.9%). Group 2 patients were younger, predominantly male, with better renal function and nutritional status (higher albumin, lower BUN). During a median follow-up of 2,057 days, 163 patients (23.5%) experienced ≥30% eGFR decline over 2 years and 119 (17.1%) initiated dialysis. Compared with Group 2 (reference), all other Groups showed significantly increased risks of both outcomes. Group 3 exhibited the highest risks for both outcomes, with adjusted HRs of 9.98 (95% CI 4.45–22.4) and 2.99 (95% CI 1.06–8.56) respectively after adjusting for confounders.

**Conclusions:**

Distinct longitudinal Hb trajectories exist in non-dialysis CKD patients and are independently associated with renal progression risk. Maintaining higher, stable Hb levels is associated with delayed renal replacement therapy initiation, whereas rapid Hb decline identifies a high-risk subgroup requiring intensive monitoring and integrated nutritional intervention.

## Introduction

Progression of chronic kidney disease (CKD) to end-stage kidney disease (ESKD) representsone of the most challenging hard endpoints in nephrology. Although proteinuria and baseline estimated glomerular filtration rate (eGFR) have been widely incorporated into risk prediction models, a considerable proportion of patients labeled as “traditionally intermediate-risk” progress rapidly to dialysis within a short timeframe, underscoring the urgent need to identify novel, dynamically monitorable, and cost-free predictive indicators to achieve more precise individualized management (1). Anemia is a common complication in CKD and is associated with disease progression (2). However, the prognostic significance of longitudinal hemoglobin trajectory patterns remains unclear, particularly in the context of the complex nutritional and metabolic derangements characteristic of CKD.

Nutritional and metabolic alterations are hallmarks of CKD progression. Protein-energy wasting (PEW), characterized by decreased body stores of protein and energy fuels, affects 18%−75% of CKD patients and is strongly associated with morbidity and mortality (3). The pathophysiology of PEW is multifactorial, involving inadequate nutrient intake due to anorexia, nausea, and dietary restrictions; hypermetabolism driven by inflammation and oxidative stress; endocrine disorders including insulin resistance and hyperparathyroidism; and nutrient losses (4). Importantly, anemia and nutritional status are intimately intertwined in CKD. Malnutrition contributes to anemia through deficiencies of hematopoietic nutrients (iron, folate, vitamin B12, protein), whereas chronic inflammation-a core component of PEW-induces erythropoietin resistance and disrupts iron metabolism via hepcidin upregulation (5). This creates a vicious cycle where nutritional deterioration exacerbates anemia, and persistent anemia further compromises nutritional status and physical function.

Hemoglobin (Hb), as the core parameter of CKD-related anemia, has traditionally been treated as a “static” cross-sectional indicator. However, Hb levels change rapidly in response to inflammation, iron metabolism abnormalities, nutritional status changes, or erythropoiesis-stimulating agent therapy. The dynamic patterns of hemoglobin may better reflect the underlying nutritional and metabolic state, as well as disease progression and treatment response, than single measurements. Previous studies have pre-dominantly focused on Hb variability in dialysis populations, suggesting that Hb fluctuations are associated with adverse outcomes (6, 7). In 2024, the French CKD-REIN cohortfirst employed group-based trajectory modeling and demonstrated that an “early rapid decline” Hb trajectory was independently associated with ESKD risk (8). However, this study was based on a European population with heterogeneous follow-up durations and irregular Hb measurement intervals. Furthermore, research on Hb trajectories specifically targeting patients with CKD stage 3 and above who have not yet initiated renal replacement therapy remains scarce. This stage represents a critical window for delaying kidney function deterioration and optimizing anemia and nutritional management; identifying distinct Hb trajectory subgroups holds significant clinical implications for early risk stratification and individualized intervention.

The present study utilizes data from the Teikyo University CKD longitudinal cohort in Japan (9) and employs feature-based clustering of longitudinal trajectories to identify distinct longitudinal Hb trajectory patterns in non-dialysis patients with CKD stages 3–4. All included patients completed a uniform 2-year follow-up period with Hb measurements scheduled at regular 90-day intervals (± 15 days), enabling precise characterization of Hb trends over time. We employ a landmark cohort design wherein Hb trajectories are constructed using measurements during a 2-year landmark period (months 0–24), and the primary predictive outcome is dialysis initiation occurring after month 24. This temporal separation ensures that exposure (Hb trajectory) temporally precedes the outcome, supporting predictive inference for the subsequent dialysis outcome, though we caution that trajectory classification remains *post hoc* and requires prospective validation. Additionally, for exploratory purposes, we examine the concurrent association between Hb trajectories and 30% eGFR decline (10, 11) occurring within the landmark period, acknowledging that this analysis is subject to temporal ambiguity and should be interpreted as hypothesis-generating rather than predictive. This dual analytical approach-distinguishing predictive modeling from exploratory association analysis-strengthens the methodological rigor and clinical applicability of trajectory-based risk stratification in pre-dialysis CKD patients.

## Materials and methods

### Study design and participants

#### Study design

This retrospective longitudinal cohort study utilized data from the Teikyo University CKD cohort, which has been previously reported (9–11). The original cohort comprised 984 CKD patients enrolled between 2002 and 2016 who received standardized clinical monitoring at the Teikyo University Hospital, including regular laboratory examinations, physical examinations, and comprehensive medication records.

#### Study population and data source

We employed a landmark cohort design (12) to address temporal ambiguity between exposure and outcome. The 2-year period from enrollment (month 0) to month 24 was defined as the landmark period, during which Hb trajectories were constructed using serial measurements. Patients who remained event-free (did not initiate dialysis or die) at month 24 constituted the landmark cohort for predictive analyses. The prediction horizon extended from month 24 to the end of follow-up (median 2,057 days from baseline; median 1,337 days from landmark). This design ensures that Hb trajectories (exposure) temporally precede dialysis initiation (outcome), supporting causal and predictive inference. For the concurrent 30% eGFR decline outcome, we acknowledge that this represents a cross-sectional association within the landmark period, where Hb trajectories and eGFR decline occur simultaneously, limiting causal interpretation.

#### Inclusion and exclusion criteria

Inclusion criteria were as follows: (1) CKD patients aged ≥18 years; (2) baseline estimated glomerular filtration rate (eGFR) between 15 and < 60 ml/min/1.73 m^2^ (CKD stages 3–4); (3) regular hemoglobin measurements scheduled at 90-day intervals (± 15 days) during the follow-up period, with at least two hemoglobin measurements available; and (4) follow-up duration ≥2 years.

Exclusion criteria were as follows: (1) receiving renal replacement therapy (hemodialysis or peritoneal dialysis) at baseline; (2) diagnosed with acute kidney injury during follow-up; (3) presence of hematological malignancies or active bleeding disorders; and (4) missing data for key variables >20%.

#### Follow-up protocol

Of 712 initially enrolled patients, 694 were included after outlier processing. All patients completed the mandatory 2-year follow-up period for hemoglobin trajectory assessment, with a median of 9 hemoglobin measurements per patient (scheduled at approximately 3, 6, 9, 12, 15, 18, 21, and 24 months). After the 2-year trajectory period, patients were followed until the initiation of renal replacement therapy, death, or administrative censoring on December 31, 2016, whichever occurred first.

### Ethical statement

This study was conducted in accordance with the Declaration of Helsinki and approved by the Institutional Review Board of Teikyo University (Review Board #14–115). Written informed consent was waived by the IRB due to the retrospective nature of the study, and all patient records and information were anonymized and de-identified prior to analysis.

### Baseline characteristics and laboratory measurements

Demographic characteristics included sex, age, body mass index (BMI), original kidney disease (diabetic nephropathy, hypertensive nephropathy, glomerulonephritis, polycystic kidney disease, solitary kidney, and others), systolic blood pressure (SBP), and hypertension status. Blood parameters included hemoglobin (Hb), white blood cell (WBC), platelet (Plt), albumin (Alb), uric acid (UA), sodium (Na), potassium (K), inorganic phosphorus (P), blood urea nitrogen (BUN), and creatinine (Cr). Blood tests were performed using a hematology autoanalyzer (Sysmex XE-5000, Kobe, Japan), and blood chemistry parameters were measured using an autoanalyzer (LABOSPECT 008, Hitachi High-Technologies Corporation, Tokyo, Japan). Serum creatinine concentration was measured by an enzymatic method. eGFR was calculated using the Modification of Diet in Renal Disease (MDRD) study equation for the Japanese population (13). CKD stage was classified based on the Kidney Disease Outcomes Quality Initiative (K/DOQI) practice guidelines (14).

### Study outcomes

The study outcomes were: (1) *a* ≥ 30% decline in eGFR from baseline during the 2-year landmark period (concurrent outcome for exploratory association analysis), and (2) initiation of renal replacement therapy (dialysis) occurring after month 24 through the end of follow-up (subsequent outcome for predictive risk stratification). 30% decline in eGFR from baseline during the 2-year landmark period, selected as a validated surrogate endpoint for CKD progression based on prior evidence that this threshold is strongly associated with subsequent kidney failure (10, 11, 15, 16) and recognized by KDIGO guidelines and the National Kidney Foundation-FDA scientific workshop as an acceptable alternative endpoint in clinical trials of CKD progression (17).

### Statistical analyses

#### General statistical methods

Continuous data are presented as mean ± standard deviation (SD) or median [interquartile range], and categorical data as number (percentage). Comparisons of baseline characteristics among thefive trajectory groups were performed using ANOVA or Kruskal–Wallis test for continuous variables, and chi-square test for categorical variables.

#### Longitudinal trajectory grouping analysis

Longitudinal trajectory grouping analysis of patients' hemoglobin levels was conducted using the R package traj (version 3.0.1), implementing the Feature-Based Clustering of Longitudinal Trajectories approach. This analysis was based on 9 follow-up measurements (scheduled at approximately 3, 6, 9, 12, 15, 18, 21, and 24 months) and followed the three-step procedure proposed by Leffondré et al. (18).

#### Feature construction

For each individual hemoglobin trajectory, we calculated 20 distinct trajectory characteristic indicators to comprehensively capture the longitudinal patterns. Trajectories with fewer than three observations were excluded from analysis. The 20 indicators included: (1) Maximum; (2) Minimum; (3) Range; (4) Mean value; (5) Standard deviation; (6) Slope of the affine approximation; (7) Intercept of the affine approximation; (8) Proportion of variance explained by the affine approximation; (9) Rate of intersection with the best affine approximation; (10) Net variation per unit of time; (11) Late variation to early variation contrast; (12) Total variation per unit time; (13) Spikiness; (14) Maximum of thefirst derivative; (15) Minimum of thefirst derivative; (16) Standard deviation of thefirst derivative; (17)First derivative's net variation per unit of time; (18) Maximum of thesecond derivative; (19) Minimum of thesecond derivative; and (20) Standard deviation of thesecond derivative. These indicators collectively characterized the magnitude, variability, trend, and curvature of each hemoglobin trajectory.

#### Dimensionality reduction and clustering

We employed spectral clustering based on Meila's algorithm for trajectory classification (19). The algorithmfirst constructed a similarity matrix S generated from a binary K-nearest neighbors (KNN) function, defined as S =(W + W^T^)/2, where the weight matrix W had elements W_ij =1 if data point j was among the K nearest neighbors of data point i, otherwise W_ij =0.

#### Optimal cluster number determination

We performed iterative clustering with cluster numbers (*k*) ranging from 2 to 8. The optimal number was determined by comprehensive evaluation of three internal validation indices (Table 1): (1) C-index (lower values indicate better separation); (2) Calinski-Harabasz index (higher values indicate better defined clusters); and (3) Wemmert-Gancarski index (higher values indicate better clustering quality). All indices were standardized to 0–1 range. *k* = 5 demonstrated the optimal balance: C-index = 0.196 (lowest among *k* = 2–5), Calinski-Harabasz = 94.886, and Wemmert-Gancarski = 0.223 (highest among *k* = 2–5), while maintaining clinical interpretability with distinct trajectory patterns. Solutions with *k* > 5 showed diminishing returns in validation indices while increasing model complexity.

**Table 1:** Internal clustering validation indices for determination of optimal cluster number.

*k*	C-index	Calinski-Harabasz	Wemmert-Gancarski
2	0.326	133.197	0.264
3	0.277	96.899	0.222
4	0.234	103.787	0.221
5	**0.196**	**94.886**	**0.223**
6	0.211	82.129	0.176
7	0.186	74.193	0.190
8	0.180	72.003	0.184

Three internal validation indices evaluated for cluster numbers k = 2 to 8.

C-index measures clustering compactness (lower = better).

Calinski-Harabasz index measures between-cluster separation relative to within-cluster dispersion (higher = better).

Wemmert-Gancarski index combines separation and compactness (higher = better).

All indices standardized to 0–1 range.

Bold values indicate optimal performance. k = 5 selected based on optimal balance across allthree indices combined with clinical interpretability.

#### Clustering stability assessment

To ensure clustering stability and reliability, the algorithm was executed with 50 random initial center iterations, and the optimal clustering result was selected. This approach minimized the impact of random initialization on final group assignments.

We explicitly acknowledge that thefive trajectory groups are identified through data-driven clustering (*post hoc*) rather than pre-defined based on clinical or biological criteria. The spectral clustering algorithm optimizes group separation based on statistical properties of the observed data, which may not replicate in independent samples. Therefore, trajectory group membership should be considered exploratory phenotypes requiring external validation, rather than established risk categories ready for clinical implementation.

#### Survival analysis

Survival curves were plotted using the Kaplan-Meier method, and differences among groups were compared using the Log-rank test. Pairwise comparisons between groups were performed, with *P*-values adjusted for multiple testing using the Bonferroni correction.

#### Exploratory association analysis

To examine the cross-sectional relationship between Hb trajectories and kidney function decline occurring simultaneously within the landmark period, Cox regression models were used to assess the association between trajectory groups and 30% eGFR decline during months 0–24. Cox proportional hazards regression was selected for the ≥30% eGFR decline outcome because this represents a fixed-timeframe endpoint (exactly 2 years) with well-defined censoring mechanisms at the landmark timepoint. This approach allows standard time-to-event analysis within the landmark period without competing risk considerations. We emphasize that this analysis is exploratory and subject to temporal ambiguity, as Hb trajectories incorporate information from the entire 2-year period while eGFR decline is assessed over the same timeframe. Results should be interpreted as hypothesis-generating associations rather than predictive relationships. Confounding factors were adjusted including sex, age, BMI, hypertension, diabetes mellitus, baseline eGFR, Albumin, C-reactive protein, 24 h urine protein, ESA use and iron supplementation.

#### Predictive risk stratification analysis

To evaluate predictive risk stratification for dialysis initiation occurring after the landmark period, we employed competing risk analyses using the Fine-Gray proportional subdistribution hazards model among patients who were dialysis-free at month 24. The Hb trajectory groups (determined from months 0–24 data) served as baseline predictors for dialysis risk during the subsequent follow-up period (month 24 to end of follow-up), with all-cause mortality prior to dialysis as the competing event. This temporal separation (exposure before outcome) supports predictive inference and risk stratification applications. Fine-Gray competing risk regression was selected for dialysis initiation occurring after the landmark period because death prior to dialysis represents a competing event that would preclude observation of the primary outcome. The subdistribution hazard model estimates the effect of Hb trajectories on the cumulative incidence of dialysis while accounting for the competing risk of mortality, providing clinically relevant risk estimates for patients who remain alive and at risk for dialysis (20). Subdistribution hazard ratios (SHR) and their 95% confidence intervals (CI) were calculated using the Fine-Gray competing risk model. Multivariable Cox regression was performed to adjust for confounding factors including sex, age, BMI, hypertension, diabetes mellitus, baseline eGFR, Albumin, C-reactive protein, 24 h urine protein, ESA use, iron supplementation and eGFR decline per year. The proportional hazards assumption was assessed using Schoenfeld residuals.

#### Software

All statistical analyses were performed using R software (version 4.3.0). Two-sided tests were used, and *P* < 0.05 was considered statistically significant.

## Results

### Patient selection and landmark cohort definition

After screening, 694 CKD patients were finally included for trajectory grouping analysis. All patients completed the mandatory 2-year follow-up period with a median of nine hemoglobin measurements per patient (scheduled at approximately 3, 6, 9, 12, 15, 18, 21, and 24 months, with median [IQR] intervals of 91 [83–98], 179 [172–187], 270 [262–278], 360 [352–368], 450 [442–457], 540 [532–548], 630 [622–637], and 721 [713–728] days from baseline). The median total follow-up duration was 2,057 [1,494–2,627] days. The mean age of patients was 64.7 ± 12.8 years, with 62.1% being male. The median baseline eGFR was 42.2 [29.6–52.3] ml/min/1.73 m^2^. The prevalence of diabetes was 41.5%, and hypertension was 87.2%.

All 694 patients completed the mandatory 2-year landmark period (months 0–24) with hemoglobin trajectory data and were dialysis-free at month 24. During the subsequent extended follow-up period (median 2,057 days from baseline; median 1,337 days from month 24), 119 patients (17.1%) initiated dialysis and 89 patients (12.8%) died prior to dialysis initiation. The landmark cohort for predictive analyses of post-landmark dialysis risk therefore comprised all 694 patients who were event-free (dialysis-free and alive) at the landmark time point (month 24).

### Hb trajectory groups during the landmark period

Based on nine follow-up hemoglobin measurements, trajectory grouping analysis identified five distinct subgroups. Group 1 (low-stable, *n* = 181, 26.1%) was characterized by persistently low hemoglobin levels with a mean of 11.8 ± 1.5 g/dL, remaining stable around 11.8 g/dl throughout the follow-up period with minimal fluctuation. Group 2 (high-stable, *n* = 157, 22.6%) maintained persistently high hemoglobin levels with a mean of 14.6 ± 1.3 g/dl, remaining stable above 14.5 g/dl throughout the observation period. Group 3 (rapid-declining, *n* = 124, 17.9%) presented with moderate baseline hemoglobin levels (~13.5 g/dl) but exhibited the most rapid decline among all groups, with a mean of 12.7 ± 2.2 g/dl and a continuous downward trajectory. Group 4 (lowest-plateau, *n* = 122, 17.6%) started with the lowest baseline hemoglobin levels (~11.0 g/dl), showed an early rapid increase to approximately 12.0 g/dl within thefirst 3–6 months, and subsequently stabilized at this low-to-moderate level throughout the remainder of follow-up (mean 12.0 ± 2.0 g/dl). Group 5 (low-declining, *n* = 110, 15.9%) started with low baseline hemoglobin levels (~12.0 g/dl), showed an early decline to approximately 11.0 g/dl, and remained persistently low throughout the follow-up period (mean 11.5 ± 2.1 g/dl).

**Figure 1:**
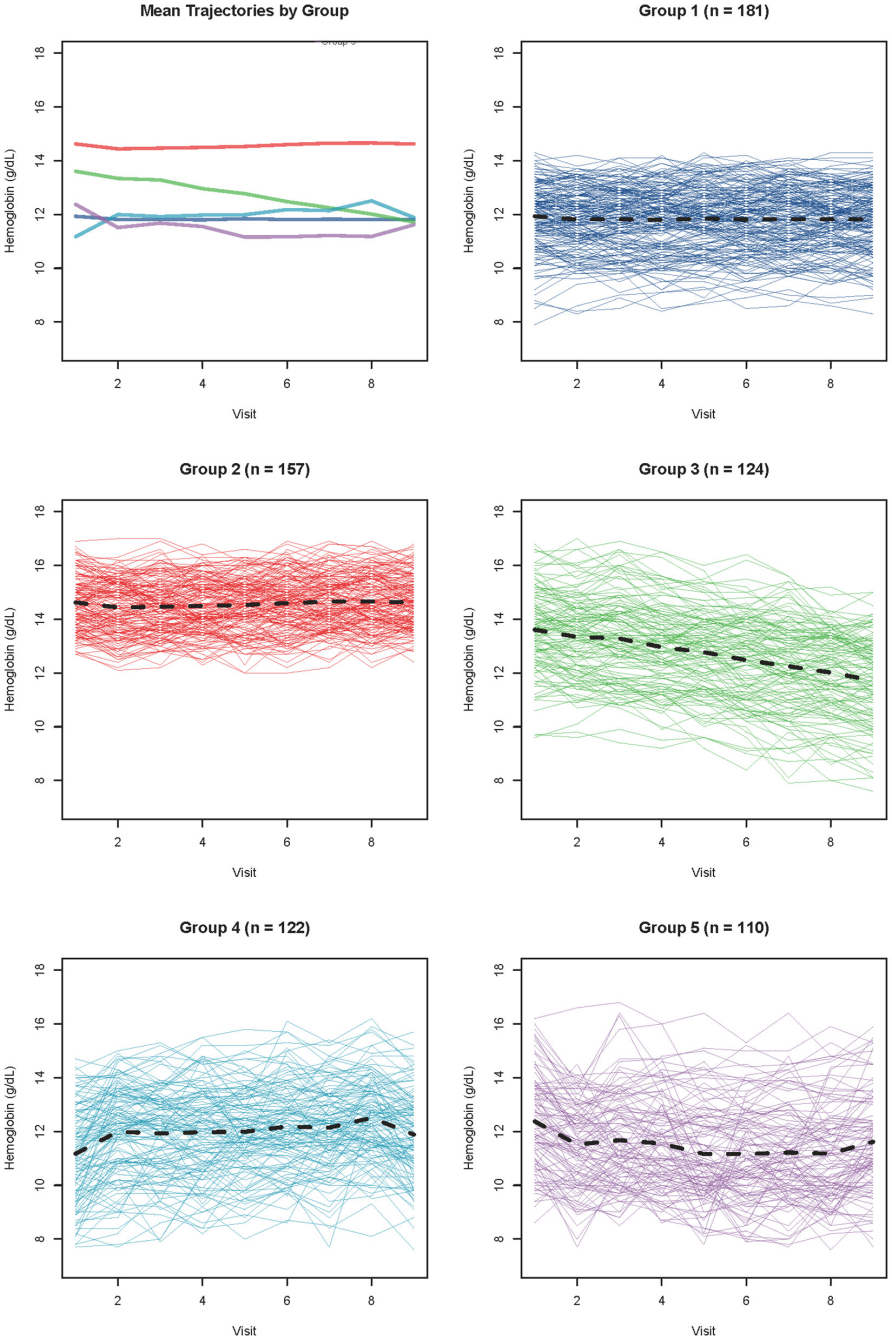
Longitudinal hemoglobin trajectory patterns in CKD stages 3–4 patients (*N* = 694).Five distinct trajectory groups identified through feature-based clustering of 9 serial hemoglobin measurements obtained at 90-day intervals over 24 months. Solid lines represent group mean trajectories; shaded areas indicate ±1 standard deviation. Y-axis: hemoglobin concentration (g/dl); X-axis: follow-up time (months). Group characteristics: group 1 (Low-Stable, *n* = 181, 26.1%, blue): persistently low Hb (mean 11.8 ± 1.5 g/dl), stable around 11.8 g/dl with minimal fluctuation; Group 2 (High-Stable, *n* = 157, 22.6%, green): persistently high Hb (mean 14.6 ± 1.3 g/dl), stable >14.5 g/dl; Group 3 (Rapid-Declining, *n* = 124, 17.9%, red): moderate baseline (~13.5 g/dl) with steepest decline (mean 12.7 ± 2.2 g/dl); Group 4 (Lowest-Plateau, *n* = 122, 17.6%, purple): lowest baseline (~11.0 g/dl), early rapid rise to ~12.0 g/dl by month 3–6, then plateau (mean 12.0 ± 2.0 g/dl); Group 5 (Low-Declining, *n* = 110, 15.9%, orange): low baseline (~12.0 g/dl), early decline to ~11.0 g/dl, persistently low (mean 11.5 ± 2.1 g/dl).

Trajectory plots (Figure 1) illustrated that Group 2 (high-stable) had hemoglobin curves significantly positioned above other groups with minimal variability throughout the observation period. Group 3 (rapid-declining) exhibited the most pronounced downward trend over time. Group 4 (lowest-plateau) and Group 5 (low-declining) both originated from severely low baseline levels but diverged in their early trajectories-early rise with subsequent plateau vs. early decline with persistent low levels, respectively. Group 1 (low-stable) maintained a consistently low but stable trajectory, positioned between the high-stable and lowest-plateau groups.

### Baseline characteristics comparison among groups

Significant differences in multiple baseline characteristics were observed among thefive trajectory groups (Table 2). Regarding demographic characteristics, Group 2 (high hemoglobin group) had the youngest patients (median 60.9 years), the highest proportion of males (82.8%), and the highest BMI (24.8 kg/m^2^). In contrast, Groups 1 and 4 were older (median 67.0 and 69.6 years, respectively) with higher proportions of females (50.8% and 47.5%, respectively). For comorbidity distribution, the prevalence of diabetes was significantly higher in Groups 3–5 (45.5%−54.5% vs. 30.6%−33.7%, *P* < 0.001), whereas the prevalence of hypertension was evenly distributed among thefive groups (*P* = 0.659). Systolic blood pressure was comparable across groups (*P* = 0.46). In terms of renal function and nutritional status, Group 2 had the best renal function with a median baseline eGFR of 50.1 ml/min/1.73 m^2^, significantly higher than other groups (*P* < 0.001). Groups 1, 4, and 5 had lower albumin levels (3.8–4.0 g/dl vs. 4.2 g/dl), suggesting poorer nutritional status; uremic toxins such as blood urea nitrogen were significantly elevated in these low hemoglobin groups (median 21.9, 22.9, and 22.8 mg/dl, respectively, vs. 17.6 mg/dl in Group 2, *P* < 0.001). Additionally, Group 5 showed the highest levels of white blood cells (median 66.0 × 10^2^/μl) and platelets (median 22.1 × 10^4^/μl), potentially indicating a higher inflammatory state. Regarding electrolyte and metabolic parameters, serum sodium, potassium, uric acid, and inorganic phosphorus levels were generally comparable across groups, with the exception of Group 5 showing slightly higher uric acid (median 6.6 mg/dl) and Group 2 having marginally higher sodium (median 141 mEq/L).

**Table 2:** Comparison of baseline characteristics across different hemoglobin trajectory groups.

Characteristics	All *N* = 694	Group 1 *N* = 181	Group 2 *N* = 157	Group 3 *N* = 124	Group 4 *N* = 122	Group 5 *N* = 110	*P*
Demographics							
Male, *n* (%)	431 (62.1%)	89 (49.2%)	130 (82.8%)	87 (70.2%)	64 (52.5%)	61 (55.5%)	<0.001
Age, years	64.7 [55.0; 72.8]	67.0 [57.5; 75.2]	60.9 [50.3; 68.1]	64.7 [54.5; 71.0]	69.6 [58.1; 74.8]	65.0 [58.5; 75.2]	<0.001
BMI, kg/m^2^	24.0 [21.5; 26.6]	23.3 [21.4; 25.5]	24.8 [23.0; 27.3]	24.7 [22.5; 26.8]	22.8 [20.8; 26.5]	23.3 [21.1; 26.5]	0.001
Systolic blood pressure, mmHg	134 [123; 148]	133 [120; 143]	132 [122; 144]	138 [128; 153]	134 [125; 151]	138 [124; 150]	0.046
Follow-up duration (days)	2,057 [1,484; 2,627]	2,165 [1,528; 2,648]	2,212 [1,633; 2,660]	2,058 [1,380; 2,607]	1,878 [1,402; 2,427]	1,974 [1,422; 2,587]	0.006
Comorbidities							
Hypertension, *n* (%)	605 (87.2%)	157 (86.7%)	132 (84.1%)	109 (87.9%)	108 (88.5%)	99 (90.0%)	0.659
Diabetes, *n* (%)	288 (41.5%)	61 (33.7%)	48 (30.6%)	63 (50.8%)	56 (45.9%)	60 (54.5%)	<0.001
Laboratory parameters							
Hemoglobin, g/L	12.8 [11.5; 14.1]	12.2 [11.2; 12.8]	14.6 [13.8; 15.3]	13.7 [12.7; 14.6]	11.2 [9.80; 12.6]	12.4 [11.1; 13.6]	<0.001
eGFR, ml/min/1.73 m^2^	42.2 [29.6; 52.3]	38.4 [27.0; 49.8]	50.1 [44.1; 54.3]	40.2 [30.1; 50.6]	35.4 [24.8; 50.3]	37.5 [26.5; 49.5]	<0.001
Albumin, g/L	4.00 [3.70; 4.20]	4.00 [3.80; 4.30]	4.20 [4.00; 4.40]	3.90 [3.70; 4.20]	3.80 [3.40; 4.10]	3.80 [3.30; 4.10]	<0.001
Total protein, g/L	7.00 [6.60; 7.30]	7.00 [6.70; 7.30]	7.10 [6.80; 7.40]	6.95 [6.50; 7.30]	6.80 [6.40; 7.20]	6.80 [6.30; 7.20]	<0.001
Uric acid, mg/dl	6.40 [5.47; 7.40]	6.30 [5.50; 7.20]	6.50 [5.50; 7.40]	6.40 [5.60; 7.40]	6.20 [5.20; 7.40]	6.60 [5.70; 7.90]	0.128
Sodium, mmol/L	141 [139; 142]	141 [140; 143]	141 [140; 143]	141 [139; 142]	141 [139; 142]	141 [139; 142]	0.007
Potassium, mmol/L	4.40 [4.10; 4.80]	4.50 [4.30; 4.8 0]	4.30 [4.00; 4.60]	4.50 [4.18; 4.80]	4.50 [4.10; 4.80]	4.50 [4.10; 4.90]	0.009
C-reactive protein, mg/L	0.09 [0.05; 0.21]	0.08 [0.05; 0.17]	0.09 [0.05; 0.19]	0.10 [0.05; 0.36]	0.11 [0.05; 0.36]	0.11 [0.05; 0.23]	0.071
Blood urea nitrogen, mmol/L	20.8 [16.0; 27.9]	21.9 [17.2; 31.0]	17.6 [14.2; 22.1]	21.2 [16.9; 27.5]	22.9 [17.4; 31.3]	22.8 [16.1; 29.3]	<0.001
24 h urine protein, mg	695 [240; 1,568]	488 [89.0; 1,176]	572 [228; 1,100]	1,235 [288; 2,183]	972 [282; 1,822]	1,076 [445; 2,004]	0.093
Medication use							
ESA use, *n* (%)	60 (8.65%)	9 (4.97%)	4 (2.55%)	8 (6.45%)	16 (13.1%)	23 (20.9%)	<0.001
Iron supplementation, *n* (%)	37 (5.33%)	6 (3.31%)	1 (0.64%)	7 (5.6%)	10 (8.20%)	13 (11.8%)	0.001

Data are presented as median [interquartile range] for continuous variables and n (%) for categorical variables.

P-values were calculated using Kruskal–Wallis test for continuous variables and chi-square test or Fisher's exact test for categorical variables.

To convert hemoglobin from g/dl to g/L, multiply by 10. To convert BUN from mg/dl to mmol/L, multiply by 0.357.

To convert creatinine from mg/dl to μmol/L, multiply by 88.4. To convert uric acid from mg/dl to μmol/L, multiply by 59.48.

BMI, body mass index; eGFR, estimated glomerular filtration rate; ESA, erythropoiesis-stimulating agent; BUN, blood urea nitrogen.

Group definitions: Group 1 (Low-Stable): persistently low hemoglobin with minimal fluctuation; Group 2 (High-Stable): persistently high hemoglobin with minimal fluctuation; Group 3 (Rapid-Declining): moderate baseline with steepest decline; Group 4 (Lowest-Plateau): lowest baseline with early rapid rise then plateaus; Group 5 (Low-Declining): low baseline with early decline then persistently low.

### Baseline characteristics at month 24 for landmark cohort

Table 3 presents the baseline characteristics of the landmark cohort at month 24, comparing clinical and laboratory parameters across thefive hemoglobin trajectory groups. At this landmark time point, significant differences were observed among groups in age, body mass index, hemoglobin levels, renal function, nutritional status, and inflammatory markers (all *P* < 0.001).

**Table 3:** Baseline characteristics at month 24 for landmark cohort.

Characteristics	All *N* = 694	Group 1 *N* = 181	Group 2 *N* = 157	Group 3 *N* = 124	Group 4 *N* = 122	Group 5 *N* = 110	*P*
Age, years	66.6 [57.0; 74.7]	69.0 [59.3; 77.2]	62.8 [51.9; 69.9]	66.6 [56.4; 73.0]	72.0 [59.9; 76.8]	66.7 [60.0; 77.0]	<0.001
BMI, kg/m^2^	23.9 [21.6; 26.6]	23.2 [21.1; 25.5]	24.8 [22.6; 27.1]	24.8 [22.5; 26.9]	22.7 [20.4; 26.5]	23.3 [21.3; 26.2]	0.001
Hemoglobin, g/L	12.6 [11.3; 14.0]	11.9 [11.0; 12.7]	14.6 [13.9; 15.3]	12.5 [11.1; 13.9]	12.1 [11.3; 13.3]	11.1 [10.1; 12.6]	<0.001
eGFR, ml/min/1.73 m^2^	37.9 [24.0; 50.1]	35.8 [22.4; 48.1]	49.6 [41.1; 55.9]	32.2 [20.6; 42.7]	33.5 [21.4; 48.4]	31.8 [17.7; 43.7]	<0.001
Albumin, g/L	4.05 [3.80; 4.30]	4.10 [3.90; 4.30]	4.30 [4.10; 4.40]	3.90 [3.68; 4.20]	3.90 [3.70; 4.20]	3.80 [3.60; 4.10]	<0.001
C-reactive protein, mg/L	0.07 [0.03,0.10]	0.06 [0.03,0.07]	0.07 [0.04,0.08]	0.08 [0.03,0.10]	0.08 [0.03,0.12]	0.10 [0.05,0.12]	<0.001
24 h urine protein, mg	4.05 [3.80,4.30]	4.10 [3.90,4.30]	4.30 [4.10,4.40]	3.90 [3.68,4.20]	3.90 [3.70,4.20]	3.80 [3.60,4.10]	<0.001

Data are presented as median [interquartile range] for continuous variables and n (%) for categorical variables.

P-values were calculated using Kruskal–Wallis test for continuous variables and chi-square test or Fisher's exact test for categorical variables.

BMI, body mass index; eGFR, estimated glomerular filtration rate.

Group 2 (High-Stable) continued to demonstrate the most favorable profile at month 24, with the highest hemoglobin levels (median 14.6 g/dl, IQR 13.9–15.3) and best-preserved renal function (median eGFR 49.6 ml/min/1.73 m^2^, IQR 41.1–55.9). These patients also maintained the highest albumin levels (median 4.3 g/dl) and lowest C-reactive protein levels (median 0.07 mg/L), consistent with their stable high-hemoglobin trajectory and superior nutritional status throughout the landmark period.

In contrast, Groups 3, 4, and 5 exhibited significantly worse characteristics at month 24. Group 3 (Rapid-Declining) showed a marked deterioration in renal function (median eGFR 32.2 ml/min/1.73 m^2^, IQR 20.6–42.7) and hemoglobin levels (median 12.5 g/dl, IQR 11.1–13.9), reflecting the progressive decline that characterized their trajectory pattern. Group 4 (lowest-plateau) and Group 5 (low-declining) demonstrated the poorest renal function at month 24 (median eGFR 33.5 and 31.8 ml/min/1.73 m^2^, respectively), with Group 5 showing the highest inflammatory burden (median C-reactive protein 0.10 mg/L).

Group 1 (Low-Stable) occupied an intermediate position, with modestly preserved renal function (median eGFR 35.8 ml/min/1.73 m^2^, IQR 22.4–48.1) and stable but low hemoglobin levels (median 11.9 g/dl, IQR 11.0–12.7). Notably, the age differences observed at baseline persisted at month 24, with Group 4 remaining the oldest (median 72.0 years) and Group 2 the youngest (median 62.8 years).

The 24-h urine protein levels at month 24 followed a similar pattern to other renal function indicators, with Group 2 showing the lowest levels and Groups 3–5 demonstrating significantly higher proteinuria. These findings at the landmark time point establish the baseline risk profile for the subsequent predictive analysis of dialysis initiation, with Group$representing the lowest-risk reference group and Groups 3–5 constituting high-risk phenotypes characterized by compromised renal function, poor nutritional status, and heightened inflammation.

### Kaplan-Meier survival analysis

**Figure 2:**
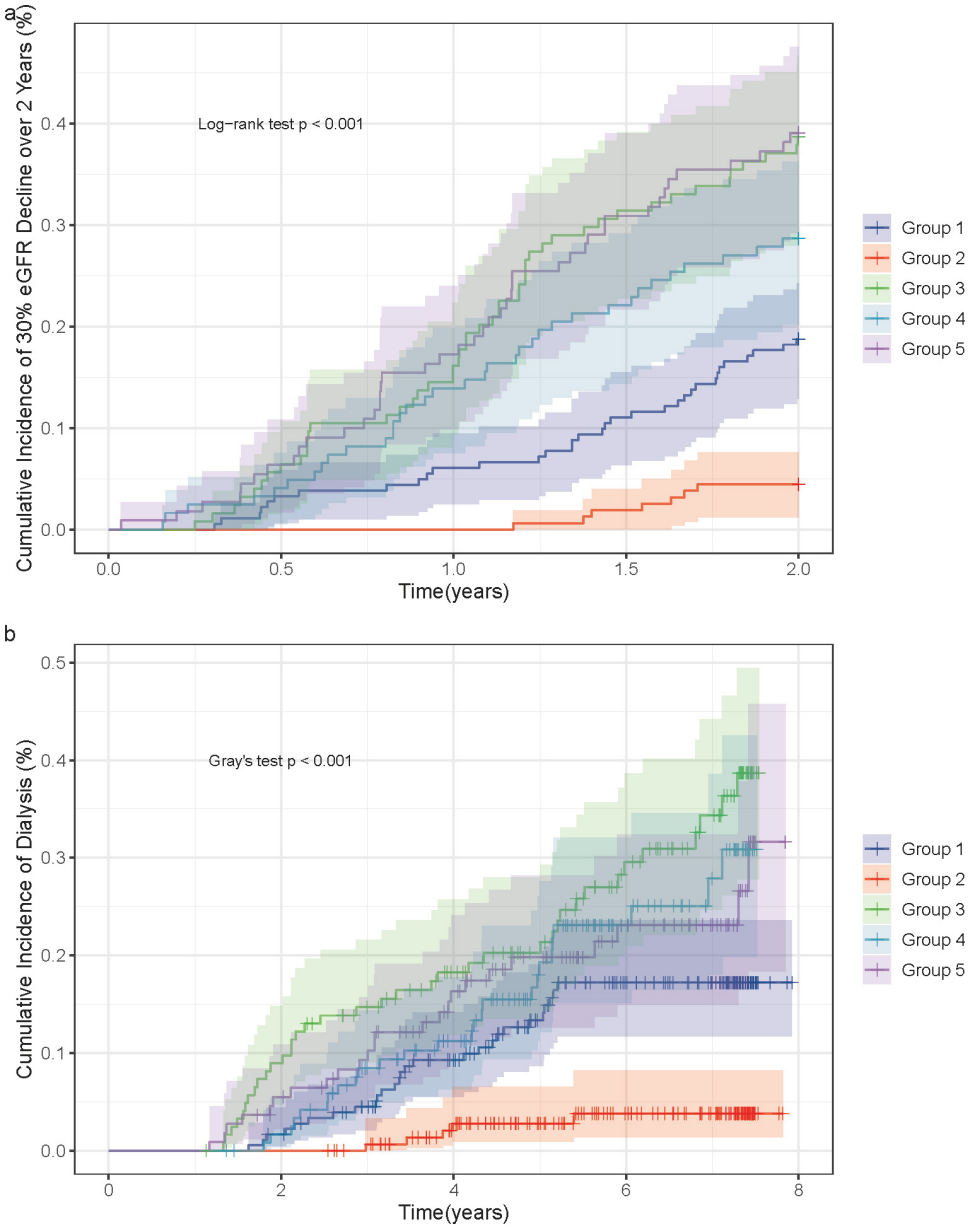
Kaplan–Meier survival curves for renal outcomes by hemoglobin trajectory groups. **(a)** Cumulative incidence of ≥30% eGFR decline from baseline during the 2-year landmark period (months 0–24). Numbers at risk shown below x-axis. Group 2 (High-Stable, green) demonstrates significantly lower event rates compared to all other groups (all *P* < 0.001). Group 3 (Rapid-Declining, red), Group 4 (Lowest-Plateau, purple), and Group 5 (Low-Declining, orange) cluster as high-risk phenotypes with statistically indistinguishable event rates (all pairwise *P* = 1.000). Group 1 (Low-Stable, blue) occupies intermediate risk position. **(b)** Cumulative incidence of renal replacement therapy (dialysis) initiation during extended follow-up after month 24 (median follow-up from landmark: 1,337 days). Death prior to dialysis treated as competing event. Group 2 maintains significantly lower dialysis risk (reference). Group 3, 4, and 5 again cluster as high-risk (pairwise *P* = 1.000), while Group 1 shows intermediate risk significantly lower than Group 3 (*P* = 0.019) but comparable to Groups 4 and 5. Log-rank *P* < 0.0001 for both panels.

During the 2-year hemoglobin trajectory period, 163 patients (23.5%) experienced a ≥30% decline in eGFR from baseline. For the renal replacement therapy outcome, the median follow-up time was 2,057 days, during which 119 patients (17.1%) initiated dialysis. Kaplan-Meier analysis demonstrated significant differences in both outcomes among thefive trajectory groups (Log-rank *P* < 0.0001 for both; Figure 2a for 30% eGFR decline over 2 years and Figure 2b for dialysis, with Group 2 (persistently high hemoglobin) consistently showing the most favorable outcomes.

In Figure 2a (30% eGFR decline over 2 years), pairwise comparisons among thefive trajectory groups revealed distinct risk stratification patterns. Compared with the persistently high hemoglobin group (Group 2), all other groups showed significantly increased risks (all *P* < 0.001). Among the lower hemoglobin trajectory groups, Group 1 (persistently low) had significantly lower risk than Group 3 (*P* < 0.001) and Group 5 (*P* < 0.001), but comparable risk to Group 4 (*P* = 0.220). Notably, Group 3, 4, and 5 exhibited similar high-risk profiles, with no significant differences detected among these three groups (all pairwise *P* = 1.000). These findings suggest a clear risk hierarchy for the short-term renal function decline outcome: Group 2 represents the lowest-risk group, Group 1 constitutes an intermediate-risk group, and Groups 3, 4, and 5 form a distinct high-risk group with comparable prognoses.

In Figure 2b (dialysis initiation), pairwise comparisons revealed nuanced differences among the trajectory groups. Similar to the 30% eGFR decline outcome, Group 2 maintained significantly lower risk than all other groups (all *P* < 0.01). However, the pattern among lower hemoglobin groups differed from the short-term eGFR outcome. Group 1 showed significantly lower risk than Group 3 (*P* = 0.019), but comparable risk to Group 4 (*P* = 0.914) and Group 5 (*P* = 1.000). Consistent with the 30% eGFR decline results, Group 3, 4, and 5 demonstrated statistically indistinguishable high risks (all pairwise *P* = 1.000). These comparisons indicate that while Group 2 remains the optimal reference group, the intermediate-risk Group 1 is distinguished from the high-risk Group 3 only in the long-term dialysis outcome, whereas Groups 3, 4, and 5 consistently cluster together as the highest-risk phenotype across both endpoints.

### Exploratory analysis: concurrent association with 30% eGFR decline

As shown in Table 4, univariate Cox regression analysis identified significantly higher risks of 30% eGFR decline in all hemoglobin trajectory groups compared with Group 2 (reference group). Group 3 (Rapid-Declining) exhibited the highest risk (HR = 10.9, 95% CI: 4.95–24.2, *P* < 0.001), followed by Group 5 (Low-Declining; HR = 11.1, 95% CI: 5.02–24.8, *P* < 0.001), Group 4 (Lowest-Plateau; HR = 7.60, 95% CI: 3.38–17.1, *P* < 0.001), and Group 1 (Low-Stable; HR = 4.53, 95% CI: 2.01–10.2, *P* < 0.001).

**Table 4:** Cox regression analysis of 30% eGFR decline over 2 years.

Parameter	Model 1			Parameter	Model 2		
	HR	95% CI	*P*		HR	95% CI	*P*
Group 1	4.53	2.01–10.2	<0.001	Group 1	3.89	1.67–9.05	0.002
Group 2		Reference		Group 2		Reference	
Group 3	10.9	4.95–24.2	<0.001	Group 3	9.98	4.45–22.4	<0.001
Group 4	7.60	3.38–17.1	<0.001	Group 4	4.23	1.81–9.92	<0.001
Group 5	11.1	5.02–24.8	<0.001	Group 5	7.21	3.13–16.6	<0.001
				Gender	1.57	1.11–2.22	0.011
				Age	0.98	0.96–0.99	<0.001
				BMI	0.99	0.96–1.04	0.803
				Hypertension	1.47	0.84–2.59	0.179
				Diabetes	1.69	1.20–2.34	0.003
				Basline eGFR	0.97	0.96–0.99	<0.001
				Albumin	0.39	0.28–0.53	<0.001
				C-reactive protein	0.79	0.61–1.02	0.066
				24 h urine protein	0.99	0.99–1.00	0.073
				ESA use	2.04	1.35–3.08	<0.001
				Iron supplementation	0.98	0.51–1.87	0.949

Model 1: univariate.

Model 2: adjusted for sex, age, BMI, hypertension, diabetes mellitus, baseline eGFR, Albumin, C-reactive protein, 24 h urine protein, ESA use, iron supplementation.

BMI, body mass index; eGFR, estimated glomerular filtration rate; ESA, erythropoiesis-stimulating agent.

After comprehensive adjustment for confounding factors including sex, age, BMI, hypertension, diabetes mellitus, baseline eGFR, albumin, C-reactive protein, 24-h urine protein, ESA use, and iron supplementation (Model 2), these associations remained statistically significant but with attenuated effect sizes. Group 3 maintained the highest adjusted risk (HR = 9.98, 95% CI: 4.45–22.4, *P* < 0.001), followed by Group 5 (HR = 7.21, 95% CI: 3.13–16.6, *P* < 0.001), Group 4 (HR = 4.23, 95% CI: 1.81–9.92, *P* = 0.001), and Group 1 (HR = 3.89, 95% CI: 1.67–9.05, *P* = 0.002). Notably, the inclusion of longitudinal nutritional and inflammatory markers (albumin, CRP) and anemia treatment variables (ESA use, iron supplementation) resulted in substantial attenuation of effect estimates, particularly for Groups 4 and 5, suggesting that these factors partially mediate the relationship between hemoglobin trajectories and concurrent kidney function decline. Additional independent predictors of 30% eGFR decline included male sex (HR = 1.57, 95% CI: 1.11–2.22, *P* = 0.011), diabetes mellitus (HR = 1.69, 95% CI: 1.20–2.39, *P* = 0.003), and lower baseline eGFR (HR = 0.97 per ml/min/1.73 m^2^, 95% CI: 0.96–0.99, *P* < 0.001), while older age was paradoxically associated with reduced risk (HR = 0.98 per year, 95% CI: 0.96–0.99, *P* < 0.001).

### Predictive model: subsequent dialysis risk after landmark

For the long-term dialysis outcome (Table 5), univariate Fine-Gray competing risk analysis similarly showed markedly elevated subdistribution hazards across all trajectory groups relative to Group 2. Group 3 (rapid-declining) demonstrated the highest unadjusted risk [subdistribution HR (SHR) = 10.9, 95% CI: 4.28–27.5, *P* < 0.001], followed by Group 4 (Lowest-Plateau; SHR = 7.87, 95% CI: 3.03–20.4, *P* < 0.001), Group 5 (Low-Declining; SHR = 7.83, 95% CI: 2.99–20.5, *P* < 0.001), and Group 1 (Low-Stable; SHR = 4.92, 95% CI: 1.90–12.8, *P* = 0.001).

**Table 5:** Competing risk fine–gray regression models of dialysis initiation for landmark cohort.

Parameter	Model 1			Parameter	Model 2			Parameter	Model 3		
	HR	95% CI	*P*		HR	95% CI	*P*		HR	95% CI	*P*
Group 1	4.92	1.90–12.8	0.001	Group 1	3.41	1.22–9.51	0.019	Group 1	1.81	0.68–4.82	0.24
Group 2		Reference		Group 2		Reference		Group 2		Reference	
Group 3	10.9	4.28–27.5	<0.001	Group 3	7.59	2.75–20.9	<0.001	Group 3	2.99	1.06–8.56	0.04
Group 4	7.87	3.03–20.4	<0.001	Group 4	2.35	0.81–6.79	0.11	Group 4	1.36	0.49–3.83	0.56
Group 5	7.83	2.99–20.5	<0.001	Group 5	3.51	1.26–9.78	0.016	Group 5	1.18	0.41–3.42	0.76
				Gender	2.37	1.48–3.81	<0.001	Gender	1.47	0.95–2.28	0.082
				Age	0.95	0.94–0.97	<0.001	Age	0.96	0.95–0.98	<0.001
				BMI	0.99	0.95–1.05	0.93	BMI	0.99	0.95–1.05	0.85
				Hypertension	2.22	1.06–4.62	0.034	Hypertension	1.78	0.87–3.66	0.11
				Diabetes	2.41	1.52–3.82	<0.001	Diabetes	2.26	1.41–3.60	<0.001
				Basline eGFR	0.92	0.89–0.95	<0.001	Basline eGFR	0.89	0.86–0.92	<0.001
				Albumin	0.32	0.22–0.45	<0.001	Albumin	0.42	0.28–0.64	<0.001
				C-reactive protein	0.99	0.97–1.02	0.74	C-reactive protein	1.01	0.99–1.02	0.58
				24 h urine protein	0.99	0.99–1.00	0.62	24 h urine protein	0.99	0.99–1.00	0.46
				ESA use	1.78	1.00–3.15	0.049	ESA use	1.37	0.85–2.20	0.19
				Iron supplementation	1.44	0.74–2.79	0.28	Iron supplementation	1.82	0.98–3.36	0.058
								eGFR decline per year	1.27	1.19–1.36	<0.001

Model 1: univariate.

Model 2: adjusted for sex, age, BMI, hypertension, diabetes mellitus, baseline eGFR, Albumin, C-reactive protein, 24 h urine protein, ESA use, iron supplementation, eGFR decline per year.

BMI, body mass index; eGFR, estimated glomerular filtration rate; ESA, erythropoiesis-stimulating agent.

In Model 2, after adjustment for baseline demographic and clinical factors (sex, age, BMI, hypertension, diabetes mellitus, baseline eGFR), nutritional status (albumin), inflammatory markers (C-reactive protein), proteinuria (24-h urine protein), and anemia treatments (ESA use, iron supplementation) from landmark cohort, the associations were substantially attenuated. Group 3 maintained the strongest adjusted association with subsequent dialysis initiation (SHR = 7.59, 95% CI: 2.75–20.9, *P* < 0.001), while Group 5 showed moderate significance (SHR = 3.51, 95% CI: 1.26–9.78, *P* = 0.016), and Group 1 retained borderline significance (SHR = 3.41, 95% CI: 1.22–9.51, *P* = 0.019). Notably, Group 4 (Lowest-Plateau) was no longer significantly associated with dialysis risk after comprehensive adjustment (SHR = 2.35, 95% CI: 0.81–6.79, *P* = 0.11), suggesting that the observed univariate association was largely confounded by the included covariates.

To address the concern that hemoglobin trajectories may primarily reflect underlying disease progression rather than act as independent predictors, we further adjusted for longitudinal kidney function trends by including eGFR decline per year as a time-varying covariate (Model 3). This adjustment resulted in further attenuation of effect estimates. Only Group 3 (Rapid-Declining) retained statistical significance (SHR = 2.99, 95% CI: 1.06–8.56, *P* = 0.04), while Groups 1, 4, and 5 were no longer significantly associated with dialysis initiation (Group 1: SHR = 1.81, 95% CI: 0.68–4.82, *P* = 0.24; Group 4: SHR = 1.36, 95% CI: 0.49–3.83, *P* = 0.56; Group 5: SHR = 1.18, 95% CI: 0.41–3.42, *P* = 0.76). These findings indicate that the Rapid-Declining hemoglobin trajectory pattern exhibits a degree of independence from concurrent disease progression markers, whereas the associations observed for other trajectory patterns were substantially explained by longitudinal changes in kidney function, nutritional status, inflammatory state, and anemia treatment patterns.

Additional independent predictors of dialysis initiation in the fully adjusted model (Model 3) included male sex (SHR = 1.47, 95% CI: 0.95–2.28, *P* = 0.082), diabetes mellitus (SHR = 2.26, 95% CI: 1.41–3.60, *P* < 0.001), lower baseline eGFR (SHR = 0.89 per ml/min/1.73 m^2^, 95% CI: 0.86–0.92, *P* < 0.001), lower albumin (SHR = 0.42 per g/dl, 95% CI: 0.28–0.64, *P* < 0.001), and more rapid eGFR decline (SHR = 1.27 per ml/min/1.73 m^2^/year, 95% CI: 1.19–1.36, *P* < 0.001). Older age remained associated with lower dialysis risk (SHR = 0.96 per year, 95% CI: 0.95–0.98, *P* < 0.001), likely reflecting more conservative treatment approaches in elderly patients.

## Discussion

### Study overview and analytical framework

This landmark cohort study employed a dual analytical approach with *post hoc* trajectory classification to examine Hb trajectories in CKD stages 3–4. For predictive risk stratification, we demonstrate that Hb trajectories identified through data-driven clustering during a 2-year landmark period are associated with subsequent dialysis initiation occurring after month 24, with rapid declining patterns showing the strongest association (adjusted HR 10.13). We explicitly acknowledge that these trajectory patterns are *post hoc* identified and require prospective validation before clinical implementation. For exploratory association analysis, we observed cross-sectional relationships between trajectories and concurrent eGFR decline within the landmark period, though we acknowledge that these concurrent associations are subject to temporal ambiguity and cannot establish directional relationships.

### Principal findings

This longitudinal cohort study identifiedfive distinct hemoglobin trajectories in non-dialysis CKD patients, revealing that dynamic trajectory patterns provide prognostic information beyond static baseline measurements, though the independence of these associations varies by trajectory pattern. Notably, Group 3, despite having moderate-to-high baseline hemoglobin levels around 13.5 g/dl, exhibited the highest risk for both 30% eGFR decline and dialysis initiation due to its rapid declining trend, with adjusted hazard ratios of 9.98 and 2.99 respectively in fully adjusted models. This finding demonstrates that declining hemoglobin trends may provide prognostic information beyond that captured by concurrent disease progression markers, though we acknowledge that residual confounding cannot be fully excluded in observational data.

### High-risk phenotypes identification

Three distinct high-risk phenotypes emerged from our analysis. Group 3, characterized by moderate baseline with progressive decline, reflected active disease progression and carried the greatest risk. Group 4, despite showing early improvement from the lowest starting point, exhibited long-term dialysis risk comparable to Group 5, suggesting that exposure to severe baseline anemia portends poor prognosis regardless of subsequent correction. Group 5 maintained continuously low hemoglobin levels with the worst baseline nutritional and renal function status. The critical observation that Group 3's risk exceeded that of Groups 4 and 5, despite superior baseline hemoglobin levels, underscores the fundamental limitation of using baseline values alone for prognostic evaluation. In contrast, Group 2 with persistently high and stable hemoglobin showed the most favorable outcomes, while Group 1 with low-moderate baseline but stable levels occupied an intermediate risk position, confirming that stable hemoglobin maintenance, even at suboptimal levels around 11.8 g/dl, is preferable to high baseline values with subsequent decline.

### Clinical and metabolic implications

This study revealsthree key clinical principles for CKD management: first, dynamic monitoring outperforms static measurements. Group 3 demonstrated that declining hemoglobin trends are more prognostically significant than absolute baseline values. Patients with moderate-to-high baseline Hb (~13.5 g/dl) who experienced rapid decline faced worse outcomes than those with stable low baseline (~11.8 g/dl), indicating that snapshot laboratory assessments may provide false reassurance when trajectories are deteriorating. Second, rapid decline trajectories identify high-risk subgroups requiring intensive intervention. The adjusted HR of 10.13 for dialysis risk in Group 3 suggests that patients showing rapid Hb decline warrant aggressive nutritional assessment and anemia management, including serial monitoring of albumin, pre-albumin, and inflammatory markers. Third, the baseline nadir effect carries lasting prognostic significance. Groups 4 and 5, despite divergent early trajectories, showed comparable long-term dialysis risk, suggesting that exposure to severe baseline anemia creates irreversible nutritional deficits and inflammatory burden not fully captured by single-timepoint measurements.

These findings support integrating longitudinal Hb slope monitoring into routine CKD care, alongside dynamic nutritional assessments as recommended by the International Society of Renal Nutrition and Metabolism (21). However, we caution that the favorable outcomes in Group 2 should not be interpreted as advocating for aggressive ESA therapy to achieve supraphysiological targets, given trial evidence that normalizing Hb to 13.0–15.0 g/dl does not reduce cardiovascular events and may increase stroke risk (22, 23).

### Practical implementation recommendations

For integration into routine CKD management, we propose: (1) Implement serial Hb monitoring every 3 months in stage 3–4 CKD patients, plotting trajectories to visually identify declining patterns; (2) For patients showing rapid Hb decline (>0.5 g/dl/year) despite adequate baseline levels, escalate to monthly monitoring and comprehensive nutritional assessment including albumin, pre-albumin, and CRP; (3) For high-risk trajectory groups (rapid decline, lowest-plateau, low-declining), consider early referral to renal dietitians and proactive anemia management, recognizing that baseline nadir effects may limit reversibility; (4) Maintain Hb stability as the primary goal rather than pursuing supraphysiological targets, given the superior outcomes of stable moderate levels vs. declining normal levels.

### Strengths and limitations

#### Strengths

Our study possesses several distinctive methodological strengths that enhance scientific rigor.

**1. Standardized measurement protocol:** Hemoglobin measurements scheduled at precise 90-day intervals enabled robust trajectory estimation and minimized information bias inherent in irregular follow-up data.

**2. Rigorous trajectory modeling:** We employed the traj package implementing Leffondré's three-step procedure, combining change indicators, principal component analysis dimensionality reduction, and *K*-means grouping with objective validity indices (18). This approach enabled identification of Group 3′s distinctive high-to-low declining pattern that time-averaged methods would misclassify as intermediate risk.

**3. Dual outcome evaluation:** Assessing both concurrent 30% eGFR decline and subsequent dialysis initiation over median 7.8 years follow-up revealed critical distinctions between short-term and long-term risk patterns, particularly the baseline nadir effect where early hemoglobin improvement did not translate to reduced long-term risk.

**4. Comprehensive pairwise comparisons:** Systematic pairwise comparisons with Bonferroni correction revealed that Groups 3, 4, and 5 clustered together as high-risk phenotypes across both endpoints, while Group 1 occupied a distinct intermediate position, nuances that simple reference-group comparisons would overlook.

**5. Robust covariate adjustment**: Comprehensive multivariable adjustment for sex, age, BMI, hypertension, diabetes mellitus, baseline eGFR, Albumin, C-reactive protein, 24 h urine protein, ESA use and iron supplementation ensured that trajectory associations captured unique pathophysiological information beyond established risk factors.

#### Limitation

Several limitations must be acknowledged in interpreting these findings.

**1. Single-center design:** The single-center Japanese cohort design may limit generalizability to other ethnic populations or healthcare systems with different anemia and nutritional management practices. Population characteristics-including anemia prevalence, ESA utilization patterns, and nutritional intervention protocols-may differ substantially in Western populations or in cohorts with different CKD etiology compositions (e.g., higher proportions of diabetic nephropathy).

**2. Incomplete etiological data:** Information on specific anemia etiologies, detailed nutritional assessments (dietary intake, body composition, pre-albumin), and lifestyle factors including smoking, alcohol consumption, physical activity levels, and dietary patterns was not systematically captured. These unmeasured confounders may be associated with both Hb trajectories and CKD progression, potentially contributing to residual confounding beyond the comprehensive clinical and laboratory adjustments we performed.

**3. Observational design constraints:** The observational design cannot establish causality, and the association between severe baseline anemia or declining trends and poor outcomes may reflect reverse causation where more advanced underlying kidney disease leads to both hemoglobin abnormalities and faster progression, rather than a direct effect of anemia itself. While comprehensive adjustment for longitudinal eGFR decline, nutritional status, inflammatory markers, and anemia treatments attenuated most associations, the Rapid-Declining pattern retained significance, suggesting partial independence from these confounders; however, unmeasured confounders (e.g., uncontrolled inflammation, subclinical infection, or unrecorded medication changes) may still contribute to residual confounding.

**4. Limited statistical power:** Our sample size of 694 patients provided limited statistical power for subgroup analyses, particularly regarding mortality outcomes.

**5. Subjective trajectory classification:** The optimal number of trajectory classes, while determined by statistical criteria and clinical interpretability, remains somewhat subjective and warrants validation in independent cohorts.

**6. Temporal ambiguity in concurrent analysis:** We explicitly acknowledge the temporal ambiguity inherent in the concurrent analysis of Hb trajectories and 30% eGFR decline within the same 2-year window. Because Hb trajectories incorporate information from the entire period, they cannot temporally precede eGFR decline occurring within that period, limiting causal and predictive interpretation. We have therefore reframed this analysis as exploratory association analysis rather than predictive modeling. In contrast, the analysis of dialysis initiation after the landmark period employs appropriate temporal separation, with trajectories serving as baseline predictors for future events. This distinction is clearly delineated throughout the manuscript to prevent over interpretation.

**7**. ***Post hoc* trajectory identification:** We explicitly acknowledge that the five trajectory groups are *post hoc* identified through data-driven clustering rather than pre-defined based on clinical or biological criteria. Prospective validation in independent cohorts is essential to assess whether these specific trajectory patterns replicate and whether they can improve upon existing risk prediction models.

## Conclusion

In summary, this landmark cohort study with *post hoc* trajectory classification demonstrates that longitudinal Hb trajectory patterns are exploratorily associated with kidney function decline within the landmark period and show predictive utility for subsequent dialysis initiation in CKD stages 3–4. The rapid declining pattern identified through data-driven clustering showed the strongest association with adverse outcome. We explicitly acknowledge that these findings are hypothesis-generating: trajectory groups are *post hoc* identified, require external validation, and should not be interpreted as established prognostic tools ready for clinical implementation. If validated in prospective studies, longitudinal Hb monitoring could potentially enhance risk stratification for timely intervention. Future research should: (1) prospectively validate these specific trajectory patterns in multi-center, multi-ethnic cohorts to assess cross-population stability of trajectory classification; (2) assess their incremental predictive value beyond established risk factors; and (3) evaluate whether trajectory-guided interventions can improve clinical outcomes.

## Data Availability

The original contributions presented in the study are included in the article/supplementary material, further inquiries can be directed to the corresponding authors.
